# The recording and characteristics of pulmonary rehabilitation in patients with COPD using The Health Information Network (THIN) primary care database

**DOI:** 10.1038/s41533-017-0058-2

**Published:** 2017-10-11

**Authors:** Ali Hakamy, Tricia M. McKeever, Jack E. Gibson, Charlotte E. Bolton

**Affiliations:** 1 Nottingham Respiratory Research Unit, NIHR Nottingham BRC, School of Medicine, University of Nottingham, Nottingham, NG5 1PB UK; 2 Division of Epidemiology and Public Health, School of Medicine, University of Nottingham, Nottingham, NG5 1PB UK

## Abstract

Pulmonary rehabilitation is recommended for patients with COPD to improve physical function, breathlessness and quality of life. Using The Health Information Network (THIN) primary care database in UK, we compared the demographic and clinical parameters of patients with COPD in relation to coding of pulmonary rehabilitation, and to investigate whether there is a survival benefit from pulmonary rehabilitation. We identified patients with COPD, diagnosed from 2004 and extracted information on demographics, pulmonary rehabilitation and clinical parameters using the relevant Read codes. Thirty six thousand one hundred and eighty nine patients diagnosed with COPD were included with a mean (SD) age of 67 (11) years, 53% were male and only 9.8% had a code related to either being assessed, referred, or completing pulmonary rehabilitation ever. Younger age at diagnosis, better socioeconomic status, worse dyspnoea score, current smoking, and higher comorbidities level are more likely to have a record of pulmonary rehabilitation. Of those with a recorded MRC of 3 or worse, only 2057 (21%) had a code of pulmonary rehabilitation. Survival analysis revealed that patients with coding for pulmonary rehabilitation were 22% (95% CI 0.69–0.88) less likely to die than those who had no coding. In UK THIN records, a substantial proportion of eligible patients with COPD have not had a coded pulmonary rehabilitation record. Survival was improved in those with PR record but coding for other COPD treatments were also better in this group. GP practices need to improve the coding for PR to highlight any unmet need locally.

## Chronic lung disease: Rolling out the rehab

Analysis of recent UK data suggests that more patients with chronic lung disease could benefit from lung rehabilitation programmes. During pulmonary rehabilitation (PR), patients with chronic obstructive pulmonary disease (COPD) work with specialists to learn exercises and optimise breathing techniques. The programmes are recommended under current guidelines, particularly for patients with a high breathlessness score. Despite this, when Charlotte Bolton and co-workers at the University of Nottingham analysed 36,189 patient primary care records gathered since 2004, they found only 9.8% of COPD patients had ever had a coded record of being assessed, referred for, or undertaken PR. Those patients who completed PR were 22% less likely to die that those who didn’t, although appeared they had also received better overall COPD care. Current smokers, those suffering from co-morbidities and younger patients were more likely to receive PR than other patient groups.

## Introduction

Pulmonary rehabilitation (PR) has become firmly established as a core management strategy in patients with chronic obstructive pulmonary disease (COPD).^1–3^ Ever since the National Institute for Health and Clinical Excellence (NICE) guidelines for COPD in the UK were issued in 2004,^4^ PR has been recommended, a finding further reiterated in the 2013 British Thoracic Society (BTS) NICE accredited pulmonary rehabilitation guidelines.^1,2^

The primary benefits of PR include improving exercise tolerance, dyspnoea, and quality of life with the strongest evidence for its role in patients with COPD and with an MRC breathlessness score of 3 or worse.^1^ The decision to refer to PR should not be based on parameters such as age or comorbidities but rather on disability and functional limitation.^1,2,5,6^ Co-morbidities in patients with COPD are recognised, impacting hugely on morbidity and mortality,^7,8^ but patients with co-morbidities gain benefit from PR and hence are not outright exclusions.^9,10^

There is increasing interest in provision of PR across the UK with the ongoing work of the National COPD audit programme: pulmonary rehabilitation workstream. Maps of PR sites have now been produced^11^ and a greater understanding of the unmet need is emerging. There remains a poor understanding of referral patterns into PR programme. A recent primary care snapshot audit of COPD in Wales, UK also confirmed low numbers of patients referred to PR.^12^

COPD is punctuated by acute exacerbations that often require hospitalisation which is known to be associated with significant mortality in COPD.^13^ A recent Cochrane review found that participating in a PR programme after an acute exacerbation reduces hospital admission and improves overall survival in patients with COPD.^14^ However, information regarding survival following the more common, usual PR is limited.^15,16^ One of the objectives of a PR programme is to improve physical activity.^1^ Increased levels of physical activity itself is beneficial for survival.^17^ We used a large UK primary care research database to identify patients with COPD, their record of pulmonary rehabilitation, demographics, and clinical characteristics. Survival rate according to pulmonary rehabilitation recording were also investigated.

## Result

### Descriptive analysis

**Fig. 1:**
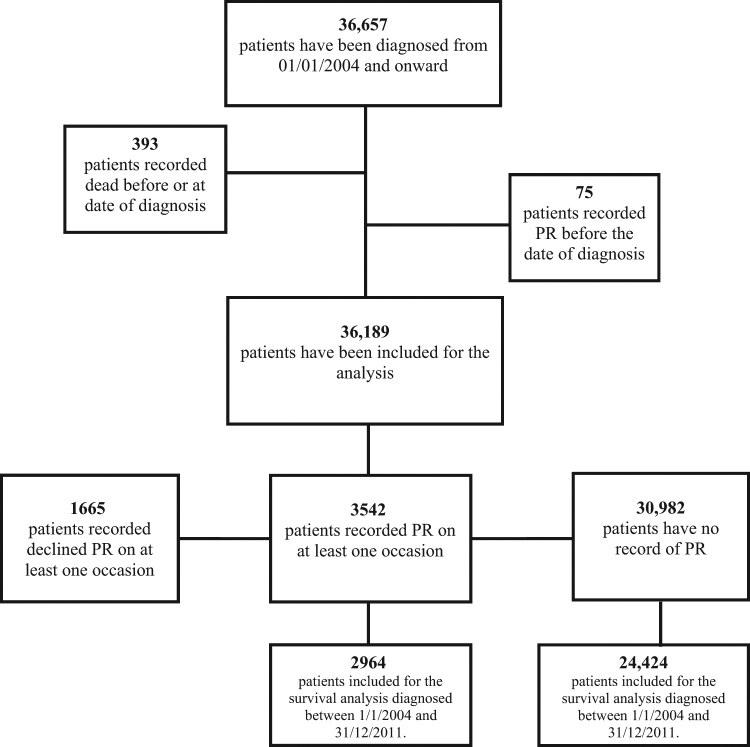
Patients with COPD from the THIN database according to pulmonary rehabilitation

Thirty six thousand six hundred and fifty seven patients had a diagnosis of COPD that was made after January 1, 2004. With further exclusions as detailed above, this resulted in a study population of 36,189 [Fig. 1]. Of these, 19,354 (53%) were male, with a mean (SD) age at diagnosis of 66.9 (11.4) years, while 18,680 (51.6%) were ex-smokers. 6602 (18.2%) patients had no record of the MRC score and 10,667 (36%) of those who had a record of MRC score, had a score of 3 or worse.

There were 3542 patients (9.8%) with a recorded PR code, 30,982 (85.6%) with no recorded PR code of any sort, and 1665 (4.6%) with a declined PR code (and there was no other code of subsequently going or being referred). Of those patients who reported MRC 3 or worse (9750 patients), 7693 (81.8%) did not have a record of PR. The median time from the date of diagnosis to the first PR record date was 875 days (interquartile range 271–1756).

### Recorded PR group to not recorded PR

In comparing the recorded PR group to the not recorded group (3542 vs. 30,982 patients) [Table 1], older patients (≥71 years old) at diagnosis were less likely to have a record of PR than younger patients (51–60 years old), with an adjusted Odds Ratio (aOR) of 0.60 (95% CI 0.53–0.76). The most deprived patients (Townsend quintile 5) were less likely to have a record of PR than the least deprived ones (Townsend quintile 1), aOR 0.61 (95% CI 0.54–0.70). Two thousand and fifty seven patients (21.1%) with MRC of 3 or worse had a record of PR. Patients with the worse MRC score were more likely to have a record of PR than those with a better score. Current smokers were more likely to have a record of PR compared with ex-smokers; aOR 2.60 (95% CI 2.41–2.86). Patients with a higher Charlson comorbidity index (four or greater) were more likely to have a record of PR than those with a lower index (1); aOR 10.44 (95% CI 9.17–12.23).

**Table 1:** Univariate and Multivariate logistic regression for patients with COPD who have recorded pulmonary rehabilitation compared with who have not recorded pulmonary rehabilitation

	Recorded PR 3542	Not recorded PR 30,982	uOR	aOR (95%CI)	*P* value
Age at the diagnosis (years)					
≤50	271 (9.86)	2477 (90.14)	0.79	0.82 (0.70–0.97)	
51–60	782 (12.07)	5695 (87.93)	1		<0.0001
61–70	1310 (11.96)	9643 (88.04)	0.98	1.01 (0.91–1.12)	
≥71	1179 (8.22)	13,167 (91.78)	0.65	0.60 (0.53–0.76)	
Gender					
Male	1849 (10.00)	16,648 (90.00)	1		0.757
Female	1693 (10.56)	14,334 (89.44)	1.06	0.99 (0.92–1.07)	
Townsend category					
I least deprived	550 (9.97)	4968 (90.03)	1		
II	577 (9.71)	5368 (90.29)	0.97	0.89 (0.78–1.02)	<0.0001
III	709 (10.20)	6245 (89.80)	1.02	0.79 (0.70–0.91)	
IV	849 (10.16)	7153 (89.39)	1.07	0.71 (0.63–0.81)	
V most deprived	779 (10.95)	6336 (89.05)	1.11	0.61 (0.54–0.70)	
Missing	78 (7.88)	912 (92.12)	0.77	0.59 (0.45–0.77)	
MRC score^a^					
MRC 1	223 (3.20)	6743 (96.80)	1		
MRC 2	880 (7.81)	10,382 (92.19)	2.56	2.41 (2.06–2.81)	<0.0001
MRC 3	1230 (20.02)	4915 (79.98)	7.56	6.43 (5.51–7.50)	
MRC 4	715 (23.99)	2266 (76.01)	9.54	8.01 (6.77–9.47)	
MRC 5	112 (17.95)	512 (82.05)	6.61	5.96 (4.58–7.76)	
Not recorded	382 (5.84)	6164 (94.16)	1.87	1.47 (1.23–1.76)	
Smoking status^a^					
Never	279 (3.24)	8324 (96.76)	0.32	0.28 (0.25–0.32)	
Ex	1696 (9.44)	16,275 (90.56)	1		<0.0001
Current	1486 (18.82)	6334 (81.18)	2.22	2.62 (2.41–2.86)	
Unknown	99 (66.89)	49 (33.11)	19.38	25.95 (17.00–39.62)	
Charlson comorbidities index					
1	304 (2.42)	12,247 (97.58)	1		
2	1494 (12.43)	10,522 (87.57)	5.72	5.40 (5.13–6.63)	<0.0001
3	920 (15.52)	5006 (84.48)	7.40	7.23 (6.64–8.75)	
≥4	824 (20.44)	3207 (79.56)	10.35	10.44 (9.17–12.23)	

^a^ For Recorded PR: MRC and smoking status was taken 15 months prior to the date of first PR record. Not recorded: it was taken 15 months prior to the index time point-875 days plus date of diagnosis

*uOR* unadjusted odds ratio, *aOR* adjusted odds ratios of recorded PR

Provision of flu vaccination and smoking cessation advice to smokers was also better in those with recorded PR than not PR: Flu vaccination: 58 vs. 48% (aOR 1.42; 95% CI, 1.31–1.53); Smoking cessation to smokers 77 vs. 52% (aOR 2.90; 95% CI, 2.52–3.34).

### Declined PR group to recorded PR

Table 2 shows the unadjusted and adjusted odds ratio of the declined PR group compared to the recorded PR group (1665 vs. 3542 patients). The patients’ age at diagnosis was associated with a code of declining PR; 71 years and older were more likely to have a record of declining PR than 51–60 years old, aOR 1.22 (95% CI, 1.03–1.46). There was no difference between those who decline PR and those with a recording for gender or the Charlson Comorbidities Index. Socioeconomic deprivation was strongly associated with recording of declining PR; the most deprived (Townsend quintile 5) patients were more likely to record declining PR than the least deprived ones (Townsend quintile 1), aOR 1.81; (95% CI, 1.46–2.25). Patients with a worse MRC score were less likely to have a record of declining PR than those with a better score. Current smokers were more likely to have a record of declining PR than ex-smokers, aOR 1.38; (95% CI, 1.21–1.58).

**Table 2:** Univariate and Multivariate logistic regression for patients with COPD who have declined pulmonary rehabilitation compared with who have recorded pulmonary rehabilitation

	Declined PR 1665	Recorded PR 3542	uOR	aOR (95%CI)	*P* value
Age at the diagnosis (years)					
≤50	113 (29.43)	271 (70.57)	0.87	0.80 (0.62–1.03)	0.007
51–60	371 (32.18)	782 (67.82)	1		
61–70	615 (31.95)	1310 (68.05)	0.98	1.04 (0.89–1.23)	
≥71	566 (32.44)	1179 (67.56)	1.01	1.22 (1.03–1.46)	
Gender					
Male	857 (31.67)	1849 (68.33)	1		0.503
Female	808 (32.31)	1693 (67.69)	1.02	1.03 (0.91–1.16)	
Townsend category					
I least deprived	179 (24.55)	550 (75.45)	1		<0.0001
II	244 (29.72)	577 (70.28)	1.29	1.36 (1.08–1.71)	
III	322 (31.23)	709 (68.77)	1.39	1.44 (1.15–1.79)	
IV	430 (33.62)	849 (66.38)	1.55	1.59 (1.29–1.96)	
V most deprived	446 (36.41)	779 (63.59)	1.75	1.81 (1.46–2.25)	
Missing	44 (36.07)	78 (63.93)	1.73	1.72 (1.13–2.60)	
MRC score^a^					
MRC 1	148 (39.89)	223 (60.11)	1		<0.0001
MRC 2	544 (38.20)	880 (61.80)	0.93	0.89 (0.70–1.29)	
MRC 3	591 (32.45)	1230 (67.55)	0.72	0.67 (0.53–0.85)	
MRC 4	279 (28.07)	715 (71.93)	0.58	0.53 (0.41–0.68)	
MRC 5	47 (29.56)	112 (70.44)	0.63	0.57 (0.38–0.85)	
Not recorded	56 (12.79)	382 (87.21)	0.22	0.21 (0.14–0.30)	
Smoking status^a^					
Never	97 (25.80)	279 (74.20)	0.83	0.84 (0.65–1.09)	<0.0001
Ex	709 (29.48)	1696 (70.52)	1		
Current	834 (36.23)	1468 (63.77)	1.35	1.38 (1.21–1.58)	
Unknown	25 (20.16)	99 (79.84)	0.60	0.70 (0.44–1.12)	
Charlson comorbidities index					
1	149 (32.89)	304 (67.11)	1		0.105
2	673 (31.06)	1494 (68.94)	0.91	0.95 (0.76–1.19)	
3	410 (30.83)	920 (69.17)	0.90	0.95 (0.75–1.20)	
≥4	433 (34.45)	824 (65.55)	1.07	1.14 (0.90–1.45)	

^a^ MRC and smoking status was taken 15 months prior to the date of first recorded or declined PR for both groups

*uOR* unadjusted odds ratio, *aOR* adjusted odds ratios of recorded decline PR

### Survival analysis

In the abridged period of COPD diagnosis for the survival analysis in order to allow follow-up time, 27,388 patients were included in this analysis: 53% were male, mean (SD) age at diagnosis was 67 (11) years, 50.5% ex-smokers, and 11% with Charlson Comorbidity Index of four or greater; which was similar to the full population. Patients with a record of PR on at least one occasion were 22% less likely to die than those with no record; adjusted hazard ratio (aHR) 0.78 (95% CI 0.69–0.88) [Table 3]. Analysing only those with an MRC of 3 or worse showed a similar survival effect, aHR 0.76 (95% CI 0.64–0.89). Survival was not affected by year of diagnosis or by GOLD airflow obstruction (where % predicted spirometry was available), data not shown. A secondary survival analysis from the date of diagnosis emphasised the above finding. Patients with recorded PR were 66% less likely to die than those with no recorded PR, aHR 0.34 (95% CI 0.31–0.39).

**Table 3:** Mutually adjusted hazard ratios of patients who have recorded pulmonary rehabilitation (*n* = 2964) to those have not recorded (*n* = 24,424)

	aHR	95% CI
Recorded PR	0.78	0.69–0.88
No record PR	1	
Age at the diagnosis (years)		
< 50	0.51	0.39–0.66
50–60	1	
60–70	1.65	1.45–1.87
> 70	3.18	2.82–3.59
Gender		
Male	1	
Female	0.78	0.73–0.83
Townsend category		
I least deprived	1	
II	0.99	0.88–1.11
III	1.08	0.97–1.21
IV	1.06	0.95–1.19
V most deprived	1.06	0.94–1.19
Missing	0.70	0.54–0.92
MRC score^a^		
MRC 1	1	
MRC 2	1.54	1.36–1.75
MRC 3	2.36	2.07–2.69
MRC 4	3.75	3.26–4.31
MRC 5	5.42	4.50–6.54
Not recorded	2.96	2.61–3.37
Smoking status^a^		
Never	0.91	0.84–0.98
Ex	1	
Current	1.18	1.07–1.29
Unknown	0.99	0.58–1.68
Charlson comorbidities index		
1	1	
2	1.13	1.03–1.23
3	1.24	1.13–1.37
≥4	1.51	1.36–1.69

^a^ For Recorded PR: MRC and smoking status was taken 15 months prior to the date of first PR record. Not recorded: it was taken 15 months prior to the index time point-875 days plus date of diagnosis

*aHR* adjusted hazard ratio, *CI* confidence interval

## Discussion

The majority of patients diagnosed with COPD have no coded record of pulmonary rehabilitation in their primary care records, even amongst those with an MRC dyspnoea score of 3 or worse. This is despite pulmonary rehabilitation being one of the most effective interventions in patients with COPD and ingrained in guidelines. Several factors appear to have influenced PR recording including younger age at diagnosis, better socioeconomic status, worse breathlessness score, current smoking status, and more comorbidities-all of which are associated with having a record of PR. An interesting survival benefit was seen among those with a record of PR compared to those without a record but PR record was associated with better coding of other COPD treatments, suggesting better package of COPD care generally.

With both the NICE COPD and the BTS pulmonary rehabilitation guidelines recommending PR to all eligible patients with COPD, the recording of PR here, in primary care records is disappointingly low.^1,2^ There are several factors that may be important to consider in relation to recording with PR, recently highlighted as crucial barriers to overcome.^18^ They include healthcare professional awareness of the referral criteria, referral pathway and the likely benefits of PR together with patient awareness and willingness to be referred and attend. Further, availability of PR and distance to travel may be further issues. The recent National COPD audit, pulmonary rehabilitation workstream in England and Wales determined spaces at each PR site and the precedent mapping exercise identified over 230 programmes and their geographical location.^11^ How that then meets the needs of the COPD population and people with other chronic respiratory disease is required, not least as PR can be repeated.

Factors that determine attendance and drop-out of patients in pulmonary rehabilitation are important to identify as good attendance facilitates the optimal functional benefits.^19–22^ Those associated with non-adherence include lower social class, social deprivation, and current smoking.^20,23,24^ However, irrespective of age,^5,6^ current smoking,^25^ or comorbidities,^10^ functional and psychological benefits can be achieved with PR and would therefore should not be barriers to referral.^1^ Indeed, PR promotes lifestyle change and as such may facilitate smoking cessation.^25^ It is thus reassuring that in our study, we report better recording of PR in those with current smoking status or with more comorbidities. One possible explanation that the recording increases with higher comorbidities index is that patients with higher comorbidities might visit their GP more frequently than lower comorbidities patients and therefore a greater opportunity for them to be referred. Additionally, these patients might feel worse due to their co-morbid illness and therefore the GP might be more likely refer them more to PR programme. Similarly, current smokers had better PR recording than others and as the PR programme often consists of smoking cessation the GP could use PR as an opportunity to facilitate and reinforce smoking cessation. However, recording was worse in those with lower socioeconomic status or older age at diagnosis. Compared to those patients who were assessed for PR in the national PR audit,^26^ the socio-demographic characteristics of this study population with record of PR in their primary care record were broadly similar.

Systematic reviews have demonstrated improved survival for patients with COPD undergoing PR after an acute exacerbation,^14,27^ such that the meta-analysis of PR following an exacerbation, the OR for mortality was 0.28; 95% CI 0.10 to 0.84.^14,27^ This was more favourable than our reported OR of 0.78 (0.69–0.88) for all recording of PR but the difference perhaps reflects the post exacerbation period of instability and generally greater increased mortality at this time. In the standard PR, delivered at clinical stability, the evidence from published literature of survival benefit is weaker as there was only data from two small randomised clinical trials where survival was a secondary outcome in both publications.^15,16^ One study compared standard PR to education only with a non-significant finding but generally indicating better survival in the PR group.^16^ The other reported better survival in those who received standard PR (92 patients) compared to a group who did not, in the following year (*P* = 0.032).^15^ Deliberately denying patients access to PR in one arm of a randomised control trial to assess survival is not an option in a government funded healthcare system like the UK, given the overwhelming evidence PR delivers in other outcomes. Moreover, although we tried to assess the impact of the most potential confounders (comorbidities, smoking status, MRC dyspnoea score), unmeasured confounders such as level of physical activity were not accounted for in the analyses. We cannot tell whether adjusting for these unmeasured confounders could account for the survival benefits.

Mechanisms as to how PR might improve mortality may be through improving physical activity.^28^ Physical activity is a strong predictor of survival in both the general population and in patients with COPD.^29,30^ Multidisciplinary PR aims to improve exercise tolerance, muscle strength, as well as psychological barriers and hence impact on physical activity.^31^ A study showed an overall improvement in the multi-composite BODE index score in patients attending PR after 3 months compared to baseline.^32^ Further, the goals of PR are a longer-term lifestyle change, that therefore likely impact further on survival.^1^ Improvements in depression and greater disease understanding from PR may also be relevant.^1^ An alternative plausible explanation for better survival that is likely is that patients with PR recorded are also receiving a more comprehensive COPD package of care in other aspects, demonstrated in the better coding for flu vaccination and smoking cessation that we used as surrogate markers here. Finally it is possible that health inequalities is a possible explanation for these findings, as patients from lower socioeconomic class tend to be less educated in general and about their disease, have less medication adherence, less access to health care than the patients from higher social class,^33,34^ and all of these factors are likely to impact survival.

A strength of this study is this primary care data is large, representative sample of patients within the UK with limited missing data. Patients with COPD were included from 2004 onwards when the Quality and Outcomes Frameworks (QOF) was established and therefore the diagnoses of COPD are more likely to be reliant on spirometry. However, the possibility of misdiagnosis of COPD remains.^35,36^ A 2004 start for this analysis also coincides with the NICE COPD guidelines being published which recommended PR for patients diagnosed with COPD with a MRC dyspnoea score of 3 or worse.^4^ Large differences exist among pulmonary rehabilitation programmes worldwide,^37^ making it is difficult to generalise wider than the UK processes of referral and healthcare delivery. Importantly, the GP research database does not record information on adherence, attendance, and core outcome measures, which can vary markedly. The use of PR Read codes in primary care data included three different codes “assessed”, “referred”, and “completed” were rarely used all together to describe a patient PR experience, generally just one of these were used. Therefore the three codes were combined to represent an episode of PR. However it is possible for a patient to be assessed or referred but not to have completed PR which would if anything move our survival results towards the null hypothesis. Although The Health Improvement Network (THIN) primary care data is used extensively for research, its primary purpose is for clinical care and it is possible the clinician discussed and recorded the PR decision as free text or had offered the PR and the patient declined it and then did not record the outcome. Both of these would bias the results towards the null hypothesis. Recording of data in general practice has been emphasised as a core competency in the Royal College of General Practitioners training curriculum, 2016.^38^ Moreover, introduction of the QOF has been suggested previously to improve the quality of recording.^39^ It is important to consider that some variables used in these analyses can change from the last recorded coded entry in records. An example of this is smoking status which when self-reported, also has limitations.. A patients’ health-related behaviour can also effect the patients participation and completion in the PR programmes.^40^ PR referral may have stemmed from hospital physicians or community teams distinct from the GP surgery.^37^ However, the entry for PR still should be recorded in the patients’ record whatever its source. And where it is not recoded, should prompt a clinician to ask at the next review and record then. It appears this population with missing data have more severe COPD as they more likely to be referred for PR than those with MRC score 1 and have a worse survival as well. The imputed anchor point that we used for the survival analysis in those who did not have PR record is a limitation but is preferable to using diagnosis date which exaggerates the survival difference-anyone who dies within a short period from diagnosis would be unlikely to have PR by this very nature.

### Clinical implication and conclusion

Despite the proven effectiveness of PR for patients with COPD, it was not recorded for the majority of patients with COPD in UK primary care records. Younger age at diagnosis, better socioeconomic status, worse breathlessness score, current smoking, and more comorbidities were associated with improved recording of PR. Further, we determined a survival benefit in favour of those with a record of PR, likely as part of a better package of COPD care. This study adds to the understanding of the characteristics of the patients with a PR record and future research needs to gain a better understanding of any barriers that prevent patients attending or being referred.

## Methods

### Data source and study population

THIN is a large nationally representative database of primary care records in the UK.^41^ Patients with COPD were extracted for the period up to May 22, 2014; patients with a new coded diagnosis of COPD made since January 1, 2004 were included. Patients were excluded whose recorded death or PR preceded recorded COPD diagnosis.

### Ethical approval

Ethical approval for the study was provided by the Cegedim Strategic Data Medical Research Scientific Review Committee (14-066). The methods were performed in accordance with relevant regulations and guidelines.

### Pulmonary rehabilitation coding

Patients were classified according to the following:

- “Recorded PR” group: at least one coding of being assessed, referred, or having completed PR records on at least one occasion; the first recording of any of these was used if the patient had more than one coding.
- “Declined PR” group, those who have only a code of declining PR.
- “Not recorded PR”: those who have no code of PR

### Demographics

The identified patient demographic variables included age at diagnosis (four categories: ≤50, 51–60, 61–70, and ≥71 years), gender, quintile of the Townsend Index of Deprivation (socioeconomic status),^42^ Medical Research Council (MRC) dyspnoea score,^43^ smoking status (current, ex, never or not recorded), and the Charlson Comorbidities Index (that preceded and included date of diagnosis of COPD divided into four categories: 1, 2, 3, or, 4 or more).^44^

There were two analyses conducted using logistic regression: a comparison of demographic and disease severity between those who have a code of recorded PR vs. those with no recorded PR, and the same characteristics between those with coded declined PR against those with recorded PR were compared. For recorded or declined PR groups, MRC score, and smoking status was used within 15 months prior to the first PR code. The median time from diagnosis with COPD to recorded PR group was determined. This was added to the diagnosis date in those patients without a recorded PR. Using this new date, MRC dyspnoea score, and smoking status were recorded from within 15 months prior to this time.

We performed a further review on two other aspects of COPD care according to recorded PR or not to put in context, namely smoking cessation coding in the current smokers and flu vaccination.

### Survival analysis

The Cox regression model was used for a multivariate survival analysis between the recorded PR and not recorded PR, adjusting for demographic, smoking status, MRC score, and level of comorbidities within the model. The start date was the date of the first PR record for recorded PR group and was the input date of diagnosis plus the median time from diagnosis to PR in the not recorded PR group. In addition, for the survival analyses, patients who did not have a PR record and had then less person-time than the median time from date of diagnosis to date of survival were excluded. The end date was the date of death, the date of leaving the dataset, or end date of data collection (May 22, 2014). Person time values were all calculated with the proper person time approach for each group. We did sensitivity analysis that excluded patients with MRC 1 and 2 and we additionally looked at survival from date of diagnosis.

All statistical analyses were performed using the Stata version 13 software (StataCorp LP, college station, TX, USA). *P*-values were determined using likelihood ratio test.

### Data availability

The Health Improvement Network (THIN) was the source for this work. Permission was granted following ethical approval by the Cegedim Strategic Data Medical Research Scientific Review Committee (14-066).
